# Combination therapy of an IL-15 superagonist complex, ALT-803, and a tumor targeting monoclonal antibody promotes direct antitumor activity and protective vaccinal effect in a syngenic mouse melanoma model

**DOI:** 10.1186/2051-1426-3-S2-P347

**Published:** 2015-11-04

**Authors:** Xiaoyue Chen, Bai Liu, Kaiping Han, Lin Kong, Terra Noel, Emily K Jeng, Sarah Alter, Mark Rubinstein, Peter R Rhode, Hing C Wong

**Affiliations:** 1 Altor Bioscience, Miramar, FL, USA; 2 Altor BioScience Corporation, Miramar, FL, USA; 3 Medical University of South Carolina, Charleston, SC, USA

## 

Cytokine-based and antibody-targeted immunotherapies have both been important approaches in the treatment of malignant cancers. However, combinational therapies of cytokines and tumor-targeting antibodies remain to be further explored, especially in advanced solid tumors. In this study, C57BL/6 mice bearing established subcutaneous B16F10 melanoma were treated with mouse melanoma targeting anti-gp75 monoclonal antibody (mAb), TA99, combined with interleukin (IL)-15 based superagonist ALT-803. This soluble protein complex consists of an IL-15 superagonist mutant (IL-15N72D) associated with an IL-15 receptor α Sushi domain - human IgG1 Fc fusion protein. Compared to native IL-15, ALT-803 possesses superior *in vivo* biologic activity for stimulating NK and CD8^+^ memory T cells. The combined ALT-803+TA99 therapy significantly exceeded either monotherapy in inhibiting melanoma tumor growth (p < 0.001) and prolonging survival (p < 0.01) of B16F10 tumor-bearing mice. Through immune cell depletion studies and immunophenotyping of peripheral cells as well as tumor-infiltrating leukocyte subsets, we found that ALT-803 enhances TA99-mediated antitumor immunity through activation of NK cells and expansion of the CD8^+^CD44^high^ memory T cell arm. In contrast, CD4^+^ T cells were shown to play more of a suppressive role in the therapeutic effect of ALT-803+TA99, possibly through involvement of regulatory T cells or ALT-803-mediated induction of PD-L1 on CD4^+^ T cells in the periphery and tumor microenvironment. Addition of anti-PD-L1 mAb to ALT-803+TA99 therapy resulted in a further increase in antitumor activity against subcutaneous B16F10 tumors. Furthermore, tumor-bearing mice that survived due to ALT-803+TA99 combination therapy exhibited long term antitumor memory against B16F10 tumor cell rechallenge. Immune-depletion studies revealed that this antitumor memory was associated with CD4^+^ T cells, CD8^+^ T cells and NK cells. Our findings suggest a therapeutic opportunity for ALT-803 in combination with tumor-targeting antibodies to simultaneously augment targeted antitumor activities of therapeutic antibodies and induce a long-term vaccinal effect which will provide durable responses in the treated host.

